# Blastic Plasmacytoid Dendritic Cell Neoplasm in an Adolescent Managed in a Resource‐Limited Setting: A Case Report

**DOI:** 10.1155/crh/1251228

**Published:** 2026-07-10

**Authors:** Garrick Laudin, Jenifer Vaughan, Sugeshnee Pather, Claudia Summers, Avishkar Maney, Atul Lakha, Muhammed Faadil Waja, Lindokuhle Goqwana, Katherine Hodkinson

**Affiliations:** ^1^ Department of Clinical Haematology, University of the Witwatersrand, Johannesburg, South Africa, wits.ac.za; ^2^ Department of Molecular Medicine and Haematology, University of the Witwatersrand, Johannesburg, South Africa, wits.ac.za; ^3^ Department of Molecular Medicine and Haematology, National Health Laboratory Service, Johannesburg, South Africa, nhls.ac.za; ^4^ Department of Anatomical Pathology, University of the Witwatersrand, Johannesburg, South Africa, wits.ac.za

**Keywords:** adolescent, blastic plasmacytoid dendritic cell neoplasm (BPDCN), case report, leukaemia

## Abstract

Blastic plasmacytoid dendritic cell neoplasms (BPDCNs) are rare, aggressive haematologic malignancies, uncommon in adolescents and often difficult to diagnose. We report a 16‐year‐old male presenting with lymphadenopathy, pancytopenia and constitutional symptoms. Bone marrow aspirate noted 84% blasts with corresponding flow cytometry and lymph node biopsy supporting the diagnosis. Peripheral blood next‐generation sequencing (NGS) revealed an *NRAS* p.(G12R) missense, gain‐of‐function mutation with a variant allele frequency (VAF) of 25%. The patient was treated with a combination of AML and ALL‐type chemotherapy and succumbed to neutropenic sepsis 259 days after his diagnosis. This case highlights the challenges in diagnosis and treatment of BPDCN in a resource‐limited setting.

## 1. Introduction

Blastic plasmacytoid dendritic cell neoplasms (BPDCNs) are rare, aggressive haematological malignancies which arise from the neoplastic transformation of nonactivated plasmacytoid dendritic cells (pDCs) (antigen presenting cells) [1].

The median age of diagnosis is 65 years with a male predominance but has been reported in children [1]. The disease typically involves the skin, peripheral blood, bone marrow, lymph nodes and central nervous system [1, 2]. BPDCN may occur either as an isolated entity or within the clinical context of an antecedent or co‐existing haematological neoplasm [3].

The biological basis of BPDCN reflects disruption of the transcriptional pathways that normally drive pDC differentiation. Commitment of haematopoietic stem cell–derived progenitors to the pDC lineage is governed by a coordinated transcriptional network. Key transcription factors regulate this process, including transcription factor 4 (*TCF4*), an E‐box regulator essential for pDC differentiation and lineage maintenance and B‐cell lymphoma/leukemia 11A (*BCL11A*), which is critical for pDC development [4, 5]. Interferon regulatory factor 8 (*IRF8*) also plays a pivotal role, directing early dendritic cell commitment in lymphoid‐primed multipotent progenitors and common dendritic progenitors; its expression is typically upregulated during this transition. BPDCN cells characteristically retain expression of *TCF4* and *BCL11A*, while *IRF8* may be mutated or aberrantly spliced, disrupting normal lineage regulation [6].

Treatment of BPDCN has traditionally been with chemotherapeutic regimens used in acute myeloid leukaemia (AML) and acute lymphoblastic leukaemia (ALL), with generally poor overall survival and a high likelihood of relapse [7]. Tagraxofusp (Elzonris) is approved as first‐line therapy for patients with BPDCN. It is a CD123‐directed therapy consisting of a fusion protein that combines recombinant human interleukin‐3 (IL‐3) with truncated diphtheria toxin and selectively targets CD123‐expressing BPDCN cells [8]. The drug has shown promise in the treatment of the disease, with drug availability and cost precluding its use in South Africa [9].

This case describes the challenges in the management of the disease in a 16‐year‐old male at Chris Hani Baragwanath Academic Hospital.

## 2. Case Presentation

A 16‐year‐old male, with no co‐morbid conditions, presented with a 3‐month history of generalised lymphadenopathy, weight loss, epistaxis and malaise.

Examination noted generalised nontender lymphadenopathy (cervical and femoral) with the largest node measuring 2 × 2 cm. The liver and spleen were nonpalpable, and no skin lesions were present on dermatologic examination.

**FIGURE 1:**
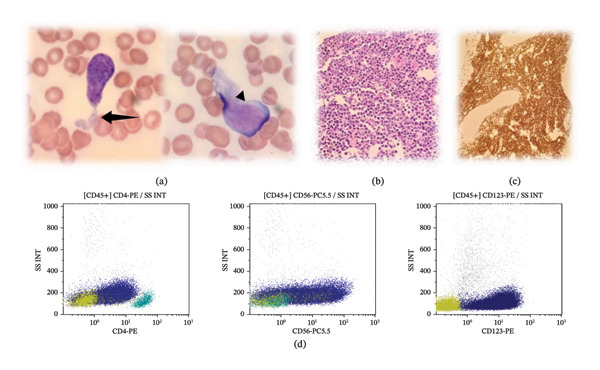
Bone marrow aspirate: (a) BPDCN tumour cells (Giemsa stain; 100 × high‐power field/HPF): medium to large cells, with an eccentric round nucleus (arrow), fine chromatin. Some forms with cytoplasmic vacuolation, distinctive tail‐like protrusions (pseudopodia) (arrowhead); bone marrow trephine biopsy: (b) extensive interstitial to diffuse infiltrate (haematoxylin and eosin/H&E; 50 × HPF); (c) tumour cells strongly positive on E‐cadherin stain (40 × HPF); flow cytometry: (d) target population on multiparameter flow cytometry (navy: tumour, yellow: lymphocytes and teal: CD4 lymphocytes).

**FIGURE 2:**
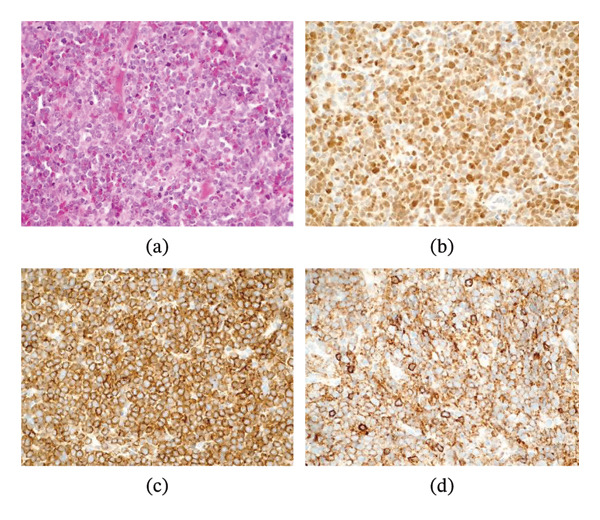
(a) Immature blastoid neoplastic cells within the lymph node (H&E, 200 × HPF); (b) diffuse nuclear TdT expression in tumour cells (IHC, 200 × HPF); (c) E‐cadherin expression in tumour cells (IHC, 200 × HPF); (d) CD4 expression in tumour cells (IHC, 200 × HPF).

The full blood count at diagnosis revealed an unbalanced pancytopenia (Table 1). Morphological examination of the bone marrow aspirate revealed 84% blastic cells with distinct morphological features noted in Figure 1a–c. Flow cytometry identified a target population of cells characterized by expression of CD123, HLA‐DR, dim CD4 and CD56 in roughly half of the cells and nuclear TdT (Table 1 and Figure 2d).

**TABLE 1:** Patient presentation laboratory and histology (bone marrow and lymph node) results.

Full blood and differential count	Result	Reference range
White cell count (× 10^^9^/L)	**0.92** (L)	3.92–10.40
Absolute neutrophil count (× 10^^9^/L)	**0** (L)	1.6–6.98
Haemoglobin (g/dL)	**9.4** (L)	13.4–17.5
Platelet count (× 10^^9^/L)	**167** (L)	171–388

Immunophenotype	Flow cytometry on bone marrow aspirate	IHC on cervical lymph node biopsy

CD4	−/dim	Membrane positivity
CD7	−/+	NT
CD33	−/+	NT
CD38	+/++	Membrane positivity
CD45	dim	NT
CD56	−/++ (on half)	Membrane positivity
CD68	NT	Focal membrane positivity
CD79a	NT	Weak membrane and cytoplasmic positivity
CD117	−/dim	NT
CD123	+/++	NT
E‐Cadherin	NT (also positive on bone marrow trephine)	Membrane positivity
HLA‐DR	++	NT
Ki67	NT	85%
Tdt	Nuclear positivity	Nuclear positivity
Negative	CD3, CD34, cCD22, cCD3, cMPO; monocytic, B‐ and platelet markers	CD8, CD34, CD35, EBER, cMPO

*Note:* Bold values highlight the abnormal result.

Abbreviations: BMA, bone marrow aspirate; c, cytoplasmic; CD, cluster of differentiation; EBER, Ebstein–Barr virus encoded RNA; IHC, immunohistochemistry; MPO, myeloperoxidase; NT, not tested; Tdt, terminal deoxynucleotidyl transferase.

Whole body computerised tomography (CT) confirmed lymphadenopathy above and below the diaphragm, with the largest lymph node measuring 2.2 cm in diameter. There was no radiographic evidence of hepatosplenomegaly on CT. Sputum nucleic acid amplification testing (NAAT) and sputum culture for *Mycobacterium tuberculosis* were negative. The lymph node architecture on biopsy was extensively effaced by diffuse sheets of blastoid cells that displayed high nuclear to cytoplasmic ratios and mitotic activity (Figure 2a). The tumour cells were immunoreactive for TdT (Figure 2b), E‐cadherin (Figure 2c), CD4 (Figure 2d), CD56, CD38 and CD79a, and the Ki‐67 proliferation index was 85% (Table 1). Chromogenic in situ hybridisation for Epstein–Barr virus encoded RNA (EBER) was negative.

Targeted next‐generation sequencing (NGS) of paired DNA and RNA was performed on the diagnostic bone marrow aspirate sample using the Ion Torrent Oncomine Myeloid Research Assay (version 5.12–5.14) (Thermo Fisher Scientific, Waltham, Massachusetts, USA) on the Ion GeneStudio S5 System (Thermo Fisher Scientific). This testing approach is routinely applied in myeloid and histiocytic neoplasms to identify RNA rearrangements and potentially targetable DNA variants. Analysis identified an *NRAS* p.(G12R) gain‐of‐function missense mutation with a variant allele frequency (VAF) of 25%. *NRAS* mutations are recurrently described in BPDCN, although they are not currently associated with established prognostic significance.

At presentation to the haematology service, ALL was initially suspected based on the context of generalised lymphadenopathy, circulating peripheral blood blast cells and the relative frequency ALL within the paediatric and adolescent population [10]. Preliminary flow cytometric analysis of the diagnostic specimen, available within 24 h, identified these cells to be CD4‐positive, raising consideration of AML with monocytic differentiation and T‐lymphoblastic leukaemia/lymphoma (T‐ALL). Subsequent immunophenotypic analyses demonstrated absence of myeloperoxidase (MPO) as well as lack of both surface and cytoplasmic CD3 expression, rendering AML with monocytic differentiation less likely and excluding T‐ALL. Strong expression of CD123 and HLA‐DR, together with partial CD56 expression, ultimately supported the diagnosis of BPDCN, which was subsequently confirmed on formal lymph node biopsy.

**FIGURE 3:**
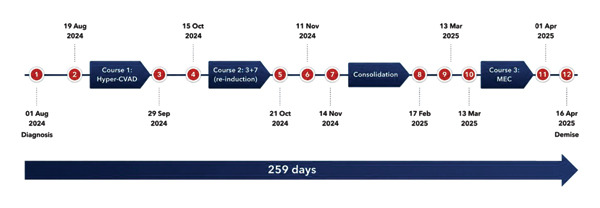
Clinical timeline of patient treatment (image created using Microsoft PowerPoint and ChatGPT [OpenAI, GPT‐5.3] based on author‐provided clinical data).

The patient received induction chemotherapy with Hyper‐CVAD (Timepoint 2, Figure 3) with 20% neoplastic cells on peripheral blood morphology assessment post‐induction (Timepoint 3, Figure 3). Reinduction was initiated with 3 + 7 (daunorubicin for 3 days and cytosine arabinoside for 7 days) (Timepoint 4 to 5, Figure 3) with no measurable residual disease (MRD) evident after reinduction (Timepoint 6, Figure 3). MRD assessment at Timepoint 6 (Figure 3) included evaluation of morphological remission, defined as the absence of morphologically identifiable BPDCN cells in the bone marrow aspirate, trephine biopsy and peripheral blood, with recovery of the peripheral counts. This was accompanied by the absence of a detectable tumour population on flow cytometry (limit of detection of 0.06 and limit of quantification of 0.1) and resolution of lymphadenopathy on clinical assessment. Consolidation chemotherapy (daunorubicin for 1 day and high‐dose cytosine arabinoside for 3 days) was administered for 4 cycles between Timepoints 7 and 8 in Figure 3. A BMAT, postconsolidation (Timepoint 9, Figure 3), revealed early relapse (∼6% neoplastic cells) on morphology and flow cytometry, with immunophenotypic evidence of clonal evolution. A decision to proceed to a salvage AML‐type reinduction (course 3) with mitoxantrone, etoposide and cytosine arabinoside (MEC) was made. MEC was administered between Timepoints 10 and 11 (Figure 3). After the third course of chemotherapy, the patient developed severe neutropenia, *E. coli* bacteraemia and succumbed to septic shock (Timepoint 12, Figure 3). His overall survival was 259 days from diagnosis.

## 3. Discussion

This case was challenging owing to the rarity of the disease in adolescent and young adults (AYA) and limited treatment options available in a resource‐limited setting.

BPDCN typically involves the blood, bone marrow, lymph nodes, central nervous system and skin. Involvement of the skin occurs in approximately 80%–90% of cases, either as the only site of involvement or accompanied by systemic disease [11–13]. Disease presentation in our patient was largely systemic, without typical skin involvement [1].

The diagnosis of BPDCN is typically based on a composite of morphology, immunophenotyping and molecular investigations. BPDCN cells are typically positive for CD123, CD4 and CD56, in addition to pDC markers (TCL‐1, TCF‐4, CD303 and CD304), and negative for lymphoid and myeloid lineage‐specific markers [14]. Owing to constraints in our resource‐limited setting, testing of additional pDC markers (TCL‐1, TCF‐4, CD303 and CD304) is unavailable in public sector laboratories.

In our patient, some nonlineage defining B‐, T‐ and myeloid associated markers were seen, as reported in approximately 4.7% of BPDCN cases. The antigens observed in our patient are documented in the literature at the following frequencies: CD7 (59%), CD33 (43%), CD117 (18%) and cytoplasmic CD79a (7%). Additional myeloid‐ and lymphoid‐associated antigens more commonly reported in BPDCN, but not detected in our patient, include CD115 (64%), CD2 (40%) and CD22 (20%) [15].

The use of molecular investigations, NGS, in our setting has becoming increasingly prevalent. NGS may assist in the differentiation of BPDCN from other myeloid neoplasms; however, mutational overlap between these entities is frequently observed [16, 17]. The mutational landscape seen in BPDCN is similar to myeloid neoplasms, with recurrent DNA variants involving the following genes: *TET2*, *ASXL1* (epigenetic regulation), *ZRSR2*, *SRSF2*, *U2AF1, SF3B1* (RNA splicing), *NRAS*, *KRAS* (signalling pathway) and *ATM* (DNA damage response) [16]. The presence of multiple DNA variants or certain variant types (e.g., *TET2* truncating mutations) correlates with poorer prognosis [16]. In a cohort study of 66 patients, *NRAS* mutations were present in 29% of those with systemic (nonskin‐only) involvement, and 0% in patients with only skin manifestations, suggesting *NRAS* mutations are linked to systemic disease, including bone marrow and extramedullary involvement [18]. NRAS mutations correlate with more aggressive, systemic phenotypes, and they have not yet been shown to independently worsen survival outcomes in the available evidence [18].

Haematopoietic stem cell transplantation (HSCT) is recommended for children with high‐risk disease features, the definition of which is currently evolving, or those in a relapsed/refractory setting where outcomes are dismal [1]. Allogeneic haematopoietic cell transplantation (allo‐HSCT) is considered the standard consolidative approach for eligible patients who achieve a first complete remission (CR1), given the aggressive nature of BPDCN and its poor prognosis with conventional chemotherapy alone. For adolescents and young adults with BPDCN, allogeneic stem cell transplantation in CR1 using myeloablative conditioning is the consensus standard for achieving durable remission and long‐term survival, with alternative donor strategies and autologous transplantation (auto‐HSCT) considered in select circumstances [19–25]. Access to allo‐HSCT in South Africa remains limited. Transplant services are largely concentrated within the private healthcare sector, placing substantial financial burden on patients. This challenge is further compounded by difficulties in identifying suitable matched unrelated donors (MUDs) for African and mixed‐race patients, owing to their underrepresentation in national donor registries [26, 27].

In general, poor outcomes with standard AML and ALL chemotherapy in BPDCN are attributable to the disease’s unique biology, high relapse risk, chemoresistance, patient‐related factors limiting tolerability and the lack of targeted therapies [15, 25, 28–32].

## 4. Conclusion

BPDCNs are rare neoplasms associated with poor outcomes to standard chemotherapy, while limited access to targeted therapies and allogeneic stem cell transplantation poses additional treatment challenges in resource‐limited settings.

## Author Contributions

Conceptualisation: Garrick Laudin, Jenifer Vaughan and Katherine Hodkinson. Study design: Garrick Laudin, Jenifer Vaughan and Katherine Hodkinson. Data collection/acquisition: Garrick Laudin, Jenifer Vaughan, Katherine Hodkinson, Sugeshnee Pather, Atul Lakha, Muhammed Faadil Waja, Lindokuhle Goqwana, Avishkar Maney and Claudia Summers. Data analysis: Garrick Laudin, Jenifer Vaughan, Sugeshnee Pather, Atul Lakha, Muhammed Faadil Waja, Lindokuhle Goqwana, Avishkar Maney and Claudia Summers, Data interpretation: Garrick Laudin, Jenifer Vaughan, Katherine Hodkinson, Sugeshnee Pather, Atul Lakha, Muhammed Faadil Waja, Lindokuhle Goqwana, Avishkar Maney and Claudia Summers. Manuscript drafting: Garrick Laudin, Jenifer Vaughan, Katherine Hodkinson, Avishkar Maney and Claudia Summers. Critical revision: Jenifer Vaughan, Katherine Hodkinson and Atul Lakha. Supervision: Katherine Hodkinson and Jenifer Vaughan. Funding acquisition: not applicable.

## Funding

This research received no special grant from any funding agency in the public, commercial or nonprofit sectors. The production of this research was made possible through employment at Chris Hani Baragwanath Academic Hospital (Department of Health, South Africa), the National Health Laboratory Service (NHLS) and the University of the Witwatersrand.

## Disclosure

All authors have read and approved the final version of the manuscript. Garrick Laudin had full access to all the data in this study and takes complete responsibility for the integrity of the data and the accuracy of the data analysis.

## Ethics Statement

Informed consent was obtained for this case report from the patient and the patient’s guardian (consent forms available on request). Ethical clearance was granted by the Human Research Ethics Committee of the University of the Witwatersrand (protocol no. **M250355**).

## Conflicts of Interest

The authors declare no conflicts of interest.

## Data Availability

Anonymised information regarding patient data will be available to the Journal upon request. No patient personal information will be shared.
